# RETRACTED: Huang et al. TPX2 Is a Prognostic Marker and Contributes to Growth and Metastasis of Human Hepatocellular Carcinoma. *Int. J. Mol. Sci.* 2014, *15*, 18148–18161

**DOI:** 10.3390/ijms27219897

**Published:** 2026-11-05

**Authors:** Yuqi Huang, Wenbin Guo, Heping Kan

**Affiliations:** 1 Department of Hepatobiliary Surgery, Nanfang Hospital, Southern Medical University, Guangzhou 510515, China; huang_yuqi2014@163.com; 2 Department of Urology, The Third Affiliated Hospital, Southern Medical University, Guangzhou 510000, China; guo_wenbin2014@163.com

The journal retracts the article “TPX2 Is a Prognostic Marker and Contributes to Growth and Metastasis of Human Hepatocellular Carcinoma” [1], cited above.

Following publication, the authors contacted the Editorial Office regarding concerns about the integrity of the cellular model used in this study [1].

In accordance with the journal’s standard procedures, the Editorial Office and Editorial Board conducted an investigation. During this process, the authors provided evidence indicating that the cell line used in the study was contaminated with a different cell type. As the validity of the experimental model could therefore not be assured, the Editorial Board concluded that the reliability of the reported findings was compromised. Consequently, the Editorial Board decided to retract this publication [1] in accordance with MDPI’s retraction policy.

This retraction was approved by the Editor-in-Chief of the *International Journal of Molecular Sciences*.

The authors have been informed of this retraction but did not provide a comment on this decision.
